# Bioinformatics strategies for lipidomics analysis: characterization of obesity related hepatic steatosis

**DOI:** 10.1186/1752-0509-1-12

**Published:** 2007-02-15

**Authors:** Laxman Yetukuri, Mikko Katajamaa, Gema Medina-Gomez, Tuulikki Seppänen-Laakso, Antonio Vidal-Puig, Matej Orešič

**Affiliations:** 1 VTT Technical Research Centre of Finland, Tietotie 2, FIN-02044, Espoo, Finland; 2 Turku Centre for Biotechnology, Tykistökatu 6, FIN-20521, Turku, Finland; 3 University of Cambridge Department of Clinical Biochemistry, Addenbrooke's Hospital, Hills Road, CB2 2QR, Cambridge, UK

## Abstract

**Background:**

Lipids are an important and highly diverse class of molecules having structural, energy storage and signaling roles. Modern analytical technologies afford screening of many lipid molecular species in parallel. One of the biggest challenges of lipidomics is elucidation of important pathobiological phenomena from the integration of the large amounts of new data becoming available.

**Results:**

We present computational and informatics approaches to study lipid molecular profiles in the context of known metabolic pathways and established pathophysiological responses, utilizing information obtained from modern analytical technologies. In order to facilitate identification of lipids, we compute the scaffold of theoretically possible lipids based on known lipid building blocks such as polar head groups and fatty acids. Each compound entry is linked to the available information on lipid pathways and contains the information that can be utilized for its automated identification from high-throughput UPLC/MS-based lipidomics experiments. The utility of our approach is demonstrated by its application to the lipidomic characterization of the fatty liver of the genetically obese insulin resistant ob/ob mouse model. We investigate the changes of correlation structure of the lipidome using multivariate analysis, as well as reconstruct the pathways for specific molecular species of interest using available lipidomic and gene expression data.

**Conclusion:**

The methodology presented herein facilitates identification and interpretation of high-throughput lipidomics data. In the context of the ob/ob mouse liver profiling, we have identified the parallel associations between the elevated triacylglycerol levels and the ceramides, as well as the putative activated ceramide-synthesis pathways.

## Background

Lipids are a diverse class of biological molecules that play a central role as structural components of biological membranes, energy reserves, and signaling molecules [1]. They are broadly defined as hydrophobic or amphipathic small molecules that may originate entirely or in part by carbanion based condensation of thioesters, and/or by carbocation based condensation of isoprene units [2]. Lipids also contribute to common pathophysiological states such as fatty liver and lipotoxic induced insulin resistance, Alzheimer's disease, atherosclerosis, and toxic manifestations of infectious diseases [3,4]. Therefore identification and characterization of these metabolic networks offers a unique opportunity to devise therapeutic strategies to prevent or reverse these pathological states.

While lipid-, and metabolome research in general, over past decades was overshadowed by the progress of genomics, recent revived and burgeoning interest in lipids that triggered several new endeavors in lipid research illustrates their critical biological importance. Lipidomics as a field aims at characterization of lipid molecular species and their biological roles with respect to the expression of proteins involved in lipid metabolism and function including gene regulation [5,6].

Several useful public resources exist representing various aspects of information on lipids, such as LIPID MAPS [7,8], Lipid Bank [9], CyberLipids [10], and LIPIDAT [11]. The LIPID MAPS consortium introduced a nomenclature that enables to represent a lipid compound by a unique 12-digit identifier [2]. LIPID MAPS also includes tandem mass spectrometry (MS/MS) fragment information for several lipid molecular species.

With the enhanced capabilities of modern MS instruments and interfaces, there has been an increase in development of global lipid analytical methods, either using liquid chromatography mass spectrometry (LC/MS) based methods focused on sensitive analyses of total lipid extracts or on specific classes of metabolites [12-15], or direct MS^n ^scanning driven by data-dependent acquisition [16-19] without chromatographic separation. Due to the structural characteristics of lipids their identification from fragment mass spectra is generally easier than for other molecular components and today's typical global lipid profiling analyses allows identifying of several hundred lipid molecular species in parallel. Informatics strategies have already been developed which utilize mass spectrometry based approaches in combination with database searches to rapidly identify specific classes of lipids, such as phospholipids [16] or PUFA-derived lipid mediators [20]. While much further progress is still needed in the area of lipid analytics, one of the biggest challenges is elucidation of biological phenomena behind the large amounts of lipidomics data currently available.

Advances in analytical methods, along with improved data processing software solutions [21-25], demand development of comprehensive lipid libraries to allow system level identification, discovery, and subsequent study of lipids. Integrative studies combining multi-tissue lipidomic profiles with other levels of biological information such as gene expression and proteomics have been made possible due to such capabilities [26,27]. Currently available databanks such as LIPID MAPS offer a necessary starting point for explorations of the lipidome and a reference for validation of results. However, in context of high-throughput lipidomic profiling and systems biology studies, the currently available online resources face threefold challenge:

1. Due to high volumes of information available from high-throughput lipidomics experiments, the database system has to be efficiently linked to the analytical platform generating the lipid profile data, as well as to chemo- and bioinformatics system for compound identification and linking the information to other levels of biological organization to enable systems approaches.

2. Due to diversity of lipids across different organisms, tissues, and cell types, it is unlikely any one database can cover all possible lipids. A mechanism is therefore necessary that facilitates identification as well as discovery of new lipid species in biological systems from available data.

3. Currently available pathway-level representation of lipids in databases such as KEGG [28] is limited to pathway representation of generic lipid classes, i.e. including mainly the head group information, and not including the fatty acid side chain information. Therefore, these lipid databases lack the level of detail that is becoming available by modern LC/MS based approaches.

Additionally, due to common structural features of different lipid classes, often regulated by the same enzymes in class-specific manner, there is a large degree of co-regulation to be expected in cellular, tissue, or biofluid lipid profiles. In order to elucidate the changes of the organism lipidome as a result of interventions, the data analysis and interpretation therefore needs to balance the analysis of global lipid pattern changes with the analysis of molecular species specific pathways.

In this paper we report a bioinformatics strategy for lipidomics analysis. We utilize the recently developed nomenclature of lipids [2] to generate a diverse scaffold of lipid compounds represented by the Simplified Molecular Input Line Entry System (SMILES) representation [29,30]. Each compound entry is linked to the available information on lipid pathways and contains the information that can be utilized for automated identification from high-throughput LC/MS-based lipidomics experiments. We investigate the changes of correlation structure of the lipidome using multivariate analysis, as well as reconstruct the pathways for specific molecular instances of interest using available lipidomic and gene expression data.

We validate our approach by investigating the lipid profiles associated with hepatic steatosis observed in ob/ob mice. Our results indicate that obesity associated hepatic steatosis involves increased liver deposition of short chain triacylglycerol species associated with proportional increase of reactive ceramide lipid species. Of interest, the contribution of triacylglycerol molecular species is heterogenesous as indicated by the presence of a subset of long triacylglycerol species that does not contribute to the development of steatosis. We also provide evidence of specific dysregulation of ceramide synthesis pathways in steatosis and the influence of gender on the liver lipid composition.

## Results and discussion

### Lipid informatics

**Figure 1:**
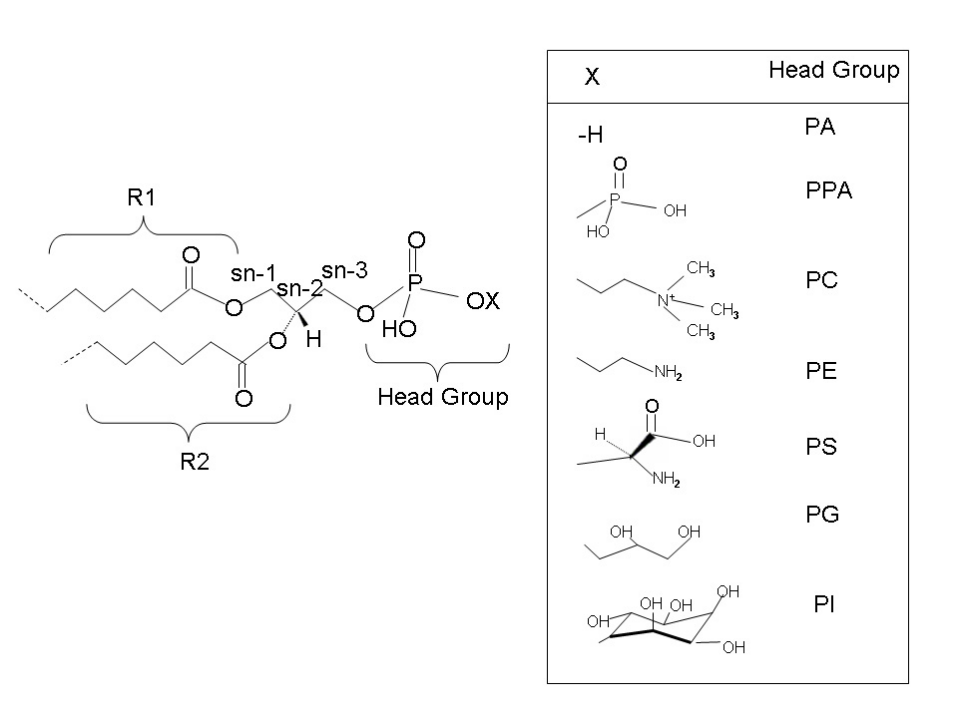
**Structures of major glycerophospholipids**. R1, R2 and X are SMILES seed variables at sn-1, sn-2 and sn-3 positions respectively. Head groups legend: PA = Phosphate, PPA = Pyrophosphate, PE = Phosphoethanolamine, PC = Phosphocholine, PS = Phosphoserine, PG = Phosphoglycerol, and PI = Phosphoinositol.

In this paper we primarily focus on studies of glycerophospholipids, sphingolipids, glycerolipids, and sterol esters. The main structural variants among these classes are variation within one or more fatty acid chains and the head group (see an example in Figure 1). In order to facilitate automated identification of lipids from lipidomics experiments, we used the structural rules of lipid molecular species to computationally generate a diverse set of lipids from "seed" fatty acids most likely to occur in living systems (Additional file 1 lists the seed fatty acids utilized in this paper). Our current choice of seeds reflects bias toward the mammalian cells, but the approach is general enough to afford suitable modifications depending on the area of interest.

The fatty acid seeds are expressed in terms of Simplified Molecular Input Line Entry System (SMILES), which is a human readable linear indexing system of atoms and bonds, dictated by specific syntax rules [29]. The modular nature of the lipid structure makes the SMILES representation very suitable for the task due to ease of algorithmic manipulation of lipid (sub)structures and their modifications. While in general multiple SMILES representations can exist for any given compound, canonical versions that enable unique SMILES representation are available. We utilize the Daylight canonical SMILES representation (Daylight, Chemical information system, Inc.). We generate a generic SMILES template for different classes of lipids and apply parsers for varying fatty acid chain lengths in order to create all possible compounds of that class in the given window of chosen fatty acid chain length. Systematic names complying with nomenclature of LIPID MAPS were generated algorithmically (Additional file 2 lists the lipid classes generated and their sizes in the database). Daylight SMILES Toolkit was tailored to get molecular weights and exact masses of compounds using elemental masses taken from literature [31].

Our approach is illustrated below using a systematic construction of glycerophospholipids classes as an example:

1. Construct generic SMILES template for glycerophospholipid class. SMILES template showing fatty acid seed variables at the sn-1 and sn-2 positions and head group at sn-3 position is:

"C(SMILES for fatty acid seed variable(R1))C(SMILES for fatty acid seed variable (R2))COP(=O)([O-])O-X)", where X represents SMILES for relevant part of head groups as shown in Figure 1.

2. Use corresponding systematic names against fatty acid seed SMILES to generate names using common name template:

"1-name of seed variable R1-2-name of seed variable R2-sn-glycero-3- name corresponding to X".

3. Convert SMILES into canonical SMILES.

4. From SMILES, obtain molecular formula and calculate molecular weight.

5. Obtain isotopic distribution of that compound and tailor it to the resolution of mass spectrometer.

The differences in length and degree of unsaturation in fatty acyl/alkyl chains lead to large diversity within each lipid class. When matching such database with the experimental lipidomics results, the searches thus inevitably result in large number of hits, both due to multiple close matches in mass as well as due to limitations of the analytical approach. In order to facilitate sifting through the multiple hits, we set up a heuristic scoring scheme based on seed fatty acid composition as described in Methods.

### Lipidomics data processing and identification

Our lipid profiling platform is based on non-targeted analysis of total lipid extracts using Ultra Performance Liquid Chromatography (UPLC) coupled to quadrupole time of flight mass spectrometry. The platform characteristics are described in detailed elsewhere [32]. In order to better understand current limitations of the analytical strategy, as well as because our computational approaches are adaptable to other platforms, including those using multiple precursor and neutral loss scanning [16,18], the analysis and data processing are described here only briefly.

**Figure 2:**
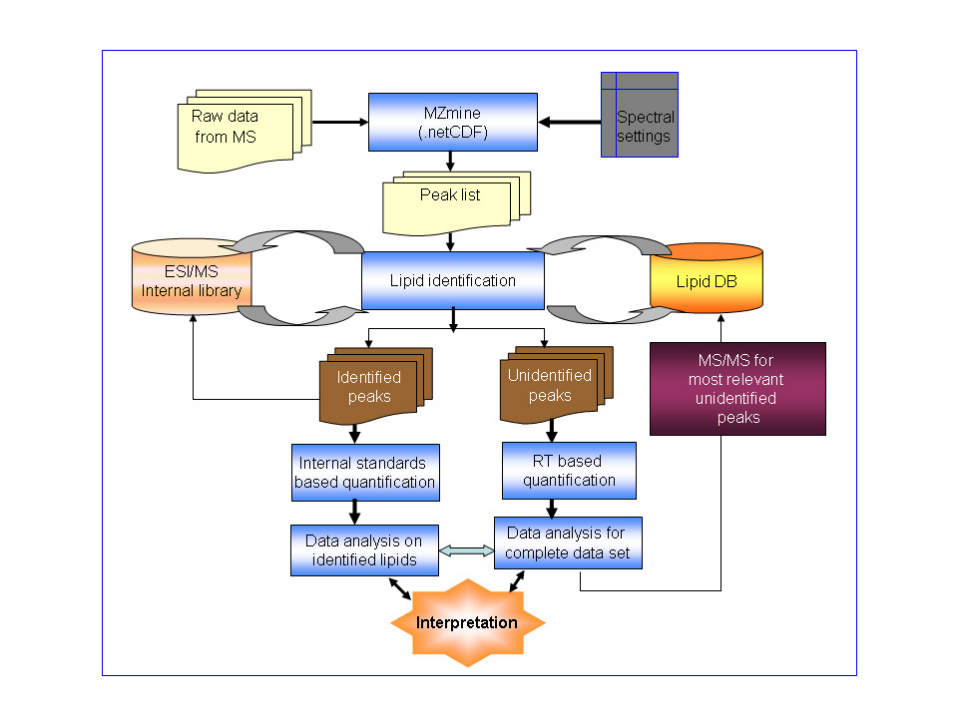
**Lipidomic platform data flow**. Summary of our lipidomic platform data flow from raw peak data to interpretation of spectra involving MZmine based data processing, lipid identification, quantification and multivariate data analysis.

An overview of the lipidomic data flow is shown in Figure 2. We convert raw mass spectrometer files to netCDF format to enable data processing with MZmine toolbox [21,22]. Peak detection and alignment parameters in MZmine are set based on preliminary investigation of platform specific characteristics such as peak shapes and retention time variation. Following the processing, each peak is characterized by mass-to-charge ratio (m/z) and retention time (RT) values.

In order to facilitate automated identification of lipids from peak lists, we compute the scaffold of theoretically possible lipids. LipidDB is a database of lipids constructed using SMILES, as described in the previous section. The internal library contains the platform-specific information about the internal standards and the lipid species identified using UPLC/MS/MS. To ease the identification of lipids based on the mass spectrometric data, we calculate isotopic distribution for every molecular species in both databases. The isotopic distribution is based on observed natural abundance of each element in the chemical formula [31]. Isotopic masses and abundances of given chemical composition are predicted using Isotope Pattern Calculator version 1.4 [33]. While the exact isotope patterns are kept in the database, the patterns are corrected for resolution of the mass spectrometer when matching with spectral data.

The internal library of lipids is searched first to ensure identification of internal standards and previously identified lipids. Retention times of these lipids are used as a constraint in lipid identification. The retention time information in part resolves the problem of identification of fatty acid moieties. The molecular species of the same class and carbon composition, but of different fatty acid composition, tend to elute at different times. The fatty acid composition can thus be determined in separate sample runs using tandem mass spectrometry (UPLC/MS/MS) in negative (phospho- or sphingolipids) or positive (acylglycerols) ion mode. In order not to compromise peak shapes in chromatographic direction, all reference UPLC/MS/MS spectra are generated in separate runs, which are set up so that ions selected for MS/MS analysis are well separated in elution time. We found the variation in retention times for the method described to be under 1.25%, as tested for multiple tissue or cell types over an extended period of time (over 18 months) for multiple UPLC C18 columns [32], therefore confirming retention time is a reliable parameter for the purpose of identification.

In the database, the redundancy due to varying fatty acid composition for the same molecular weight can be represented using the common notation showing total number of carbons and double bonds. For example, a diacylglycerophosphocholine species GPCho(16:0/20:4(5Z,8Z,11Z,14Z)) (named as 1-hexadecanoyl-2-(5Z,8Z,11Z,14Z-eicosatetraenoyl)-sn-glycero-3-phosphocholine using LIPID MAPS nomenclature) could be represented also as GPCho(36:4). However, GPCho(36:4) could also represent other molecular species, for example GPCho(20:4(5Z,8Z,11Z,14Z)/16:0) or GPCho(18:2(9Z,12Z)/18:2(9Z,12Z)).

Peaks not identified by internal library are searched in LipidDB. Lipid identifications with LipidDB involve matching m/z, comparing RT range (based on knowledge on lipids from internal library), checking heuristic score and/or MS/MS. Matching of m/z value is a pre-requisite for identification. In some cases, isobaric species are distinguished based on retention time ranges and MS/MS. Protonated phosphocholine species are identified at even m/z and sphingomyelin species are identified at odd m/z values. We also check if identifications originate from the isotopic masses at the same retention time. Ultimately, identification of isobaric species, if not separated chromatographically, also depends on the mass resolution and type of the mass spectrometer. Specifically, we have observed co-fragmentation using UPLC/MS/MS in phosphatidylcholines and ethanolamine plasmalogens in a few instances. In such cases, instruments with MS^n ^capability and high resolution detectors (*i.e*., Orbitrap or FTMS) may be necessary for exact identification.

### Reconstruction of lipid molecular pathways

Following lipidomics data processing and identification, data analysis usually includes exploration of data as well as of their putative biological meaning. In addition to the level changes of specific metabolites, which can be analyzed using univariate statistical approaches, co-regulation of metabolites is also of interest. The interdependence of metabolites is driven by the underlying biophysical mechanisms such as chemical equilibrium, mass conservation, or asymmetric control distribution [34]. Since the lipids of the same class may be in part regulated by the same enzymes, high degree of within-class co-regulation is to be expected. Correlation network analysis has proved to be a valuable tool for exploring and visualizing co-regulations in metabolomics data [26,35,36]. A matrix of correlation coefficients, an indirect measure of distance between metabolites [37], is computed using pair-wise correlation between the corresponding concentrations of lipids in a given sample. The matrix is visualized in the form of metabolite correlation network based on a certain threshold criteria over correlation coefficient values.

In order to gain insight into the molecular mechanisms underlying the observed co-regulation (or similarly for de-regulation in specific context), the clustered lipids need to be mapped into the known pathways. Kyoto Encyclopedia of Genes and Genomes (KEGG) [22] has been the main source of information on metabolic pathways. However, KEGG lipid pathway representation is generally limited to generic lipid classes, *i.e*., including mainly the head-group information, and not including the fatty acid side-chain information. As the level of information from MS studies is specific instance of subclass (*e.g*., 1-octadecanoyl-2-dodecanoyl-sn-glycero-3-phosphocholine) and not the common sub class itself (*e.g*., 1-acyl-2-acyl-sn-glycero-3-phosphocholine), a mechanism is necessary to convert generic enzymatic and pathway information from KEGG database to a specific instance under study. As we have implemented LIPID MAPS nomenclature, conversion of KEGG generic names into LIPID MAPS common subclass names and in turn to specific instance names allows mapping of identified lipids into pathways directly from MS-based studies with other levels of information.

**Figure 3:**
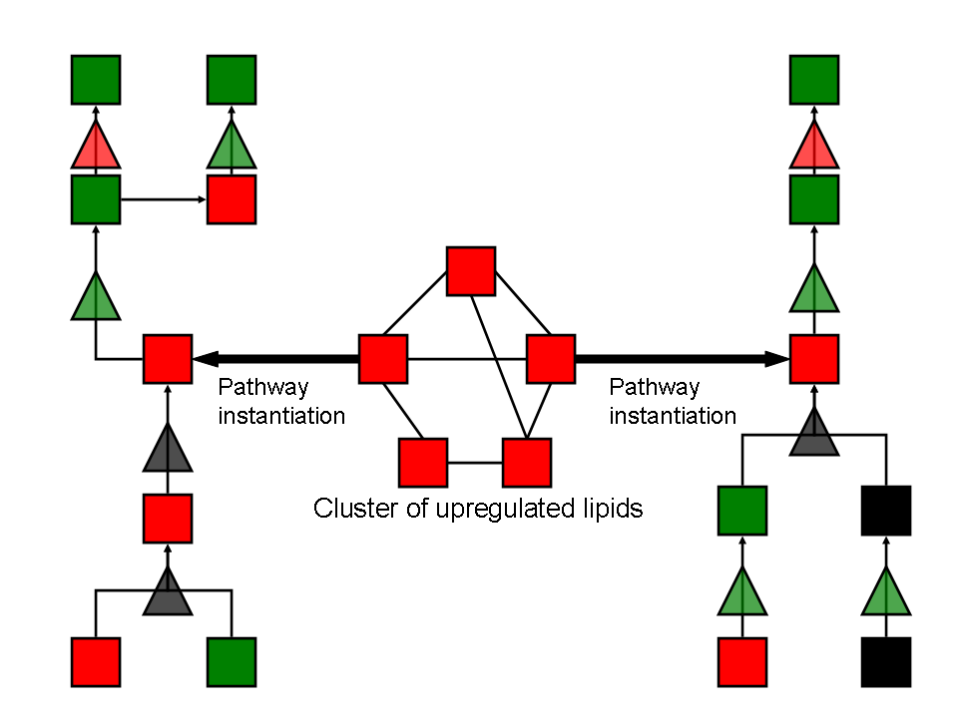
**Lipid pathway instantiation**. An illustrative example of instantiation of two co-regulated molecular species in the context of known lipid pathways. Upregulated species and enzymes (triangles) are in red, downregulated in green, unchanged in black.

We solve the limitation of generic lipid pathways by instantiating KEGG (or related) pathways for specific lipid molecular species of interest (Figure 3). In practice, our strategy to represent KEGG pathways involves the following steps:

1. Convert generic names of lipids in the KEGG reference lipid pathway into systematic common subclass names which enable to convert into systematic name for particular lipid as per LIPID MAPS consortium.

2. Construct XML schema to represent lipid pathway with systematic names of lipids and known EC numbers.

3. Generate XML document for a queried lipid.

4. Use megNet pathway visualization tool [38] to display the correlation network of lipids linked to pathways and ontologies.

Such approach affords visualization of pathways of interest in the context of observed biological data, including data from other levels such as microarray experiments. Presently we have not added additional level of quantitative analysis based on instantiated pathway information, but this is one of future considerations. One should bear in mind the complexity of such challenge as lipids are regulated systemically and their levels reflect complex systemic balance, therefore their pathways generally involve multiple tissues and complex dynamics [39].

### Lipid profiling of liver tissue in an obese mouse model

**Figure 4:**
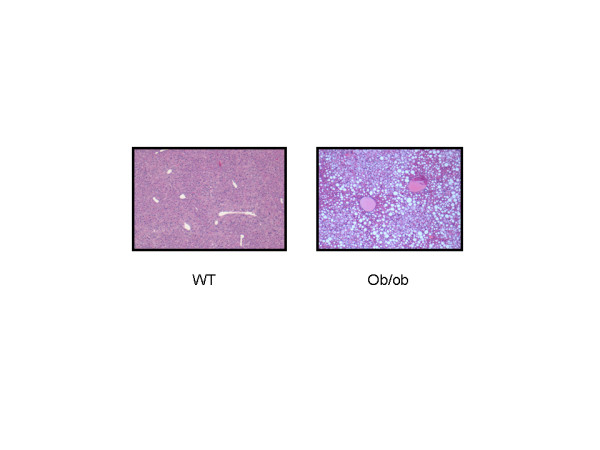
**Wild type and ob/ob mice liver cells**. Liver tissue images of wild type (WT) and ob/ob mice.

We illustrate the combined informatics and analytical approach on the liver of ob/ob mice. The ob/ob is an obese, insulin resistant mouse model resulting from the spontaneous mutation of the *ob *gene encoding the leptin protein [40]. The ob/ob mouse is commonly studied as a model for early onset of severe obesity, insulin resistance and fatty liver. Figure 4 shows typical liver tissue images of ob/ob and wild type (WT) mouse, respectively. Lipid droplet accumulation is clearly seen in the obese model. Obesity is associated with deposition of triacylglycerols (TGs) in the liver tissue (hepatosteatosis). Fatty liver develops as a result of increased free fatty acid (FFA) availability in the context of obesity and insulin resistance associated to increased hepatic glucose production [1]. Elevated hepatic FFA levels, which further lead to increased esterification into TGs, may result from the combined effect of increased influx of plasma FFAs, increased *de novo *FFAs, and decreased β-oxidation [41].

**Figure 5:**
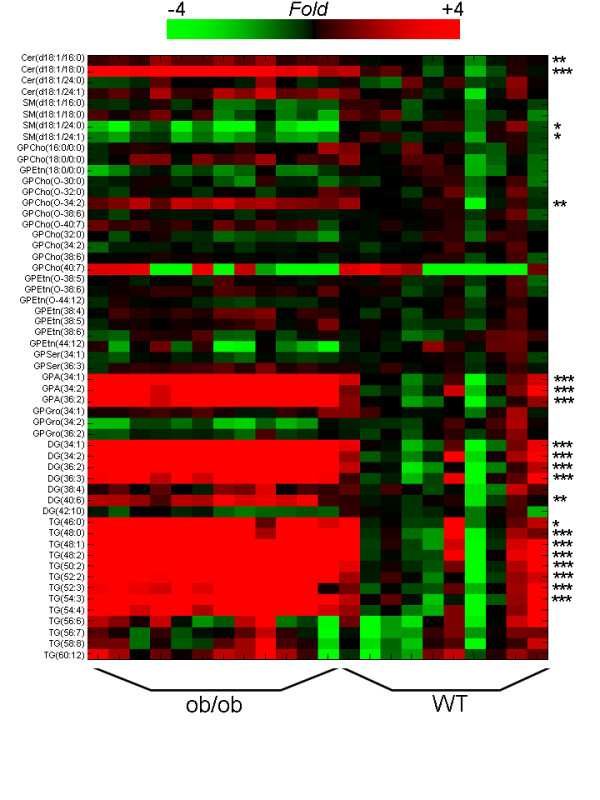
**Selected liver lipid profiles from the ob/ob mouse model**. Array view of the lipid profiles. The changes are relative to the median intensity of individual molecular species within the Wild Type group. The *p*-values were calculated based on two-sided *t*-test, conservatively adjusted by a Bonfferoni correction for the total number of 192 identified lipids: *(*p *< 0.05), **(*p *< 0.01), ***(*p *< 0.001).

The following genotypes were used for analysis: Wild Type (WT) and ob/ob. The study included 12 ob/ob (6 male, 6 female) and 10 WT (7 male, 3 female) mice of 16-week age. Figure 5 lists the results of ULPC/MS lipidomic profiling for selected molecular species, out of total 192 identified molecular species. Notable changes are upregulation in the ob/ob livers of tri- and di-acylglycerol species, diacylphosphoglycerols as well as specific reactive ceramide species. Sphingomyelins, the substrate for ceramide synthesis, appeared downregulated in the liver of the ob/ob mice compared to their lean littermates. The increase of acylglycerols should therefore be considered the hallmark leading to the development of the fatty liver observed in the ob/ob mice [42,43].

**Figure 6:**
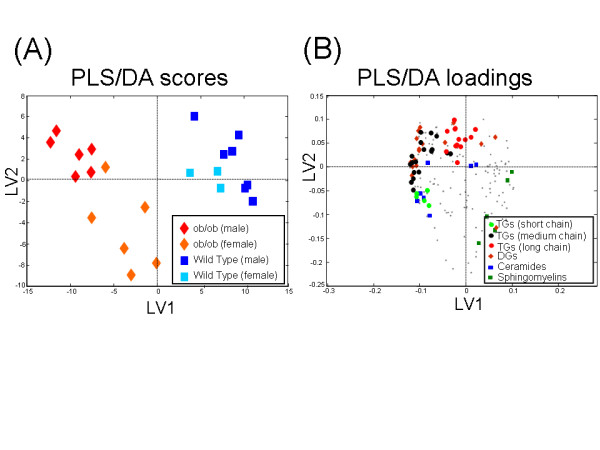
**PLS/DA analysis of the ob/ob mouse model**. (A) Score plot reveals genotype differences, as well as gender specific differences. (B) Loadings reveal major lipid classes associated with genotype differences.

In order to include the correlation structure of lipidomics data into the analysis and therefore explore possible associations between different lipid molecular species, we applied the partial least squares discriminant analysis (PLS/DA) [44,45] using the SIMPLS algorithm to calculate the model [46]. PLS/DA is a common approach to multivariate metabolomics data analysis [47,48]. PLS analysis maximizes the product of variance matrix of measured variables (e.g. lipid profile data) and correlation of measured data with properties of interest (e.g. ob/ob and WT groups). Venetian blinds cross-validation method [49] (with 4 splits) and *Q*^2 ^scores were used to optimize the model. Two latent variables were included in the model with the *Q*^2 ^= 58%, which can be considered as a significant model. Figure 6A shows the score plot of the PLS/DA model, with as expected clear separation between the genotypes.

The loadings in Figure 6B indicated that the observed separation is largely due to accumulation of acylglycerols in ob/ob mouse livers. Of interest most of the ceramides (including the most abundant Cer(d18:1/18:0) and Cer(d18:1/16:0) species) correlated with the short chain triacylglycerols, suggesting accumulation of reactive ceramide species increase in the liver of the ob/ob mice proportionally to the accumulation of triacylglycerol levels. Curiously, similar correlation between ceramides and triacylglicerols was lost when considering the pool of long chain triacylglycerols. Additionally, we observed the separation of lipid profiles based upon gender basis. The correlation between triacylglycerols and ceramides is particularly interesting since reactive ceramide species are believed to play an important role in development of obesity associated insulin resistance [50]. Therefore our results suggest that measurement of triacylglycerol in liver may be a good indirect indicator of other reactive lipid species pathogenically relevant for the development of insulin resistance.

**Figure 7:**
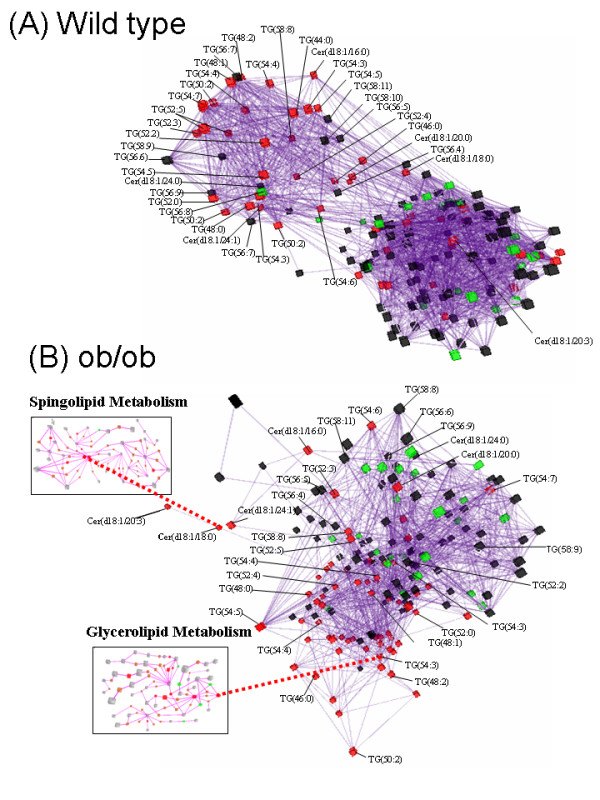
**Correlation networks for ob/ob and WT mouse liver lipid profiles**. Selected co-regulated cluster of lipid molecular species, including ceramides and acylglycerols. This network is based on Pearson correlation coefficient, r > 0.75 and statistic p-value < 0.001. Colored nodes in red (up regulation), green (down regulation) and black (no change) are based on 1.5-fold change cut off on mean value comparisons for ob/ob *vs*. WT mice. (A) Correlation network for WT mouse liver lipidomic data (B) Correlation network for ob/ob mouse liver lipidomic data.

We also investigated linear associations among lipid species by generating a correlation network. In the network, edges between the nodes representing lipid species are drawn if the Pearson correlation meets the cutoff criterion (*r *> 0.75 and *p*-value < 0.001). The nodes are colored based on fold change values comparing the mean lipid levels of obese and WT mice. Interestingly, the network corresponding to WT mouse liver sample contains almost double the number of edges (2073) as compared to the number of edges (1055) in the ob/ob mouse liver sample network. Selected clusters of co-regulated lipids corresponding to wild type and ob/ob mouse liver samples are shown in Figure 7. The observed decrease in the number of correlations among the lipid species under ob/ob condition as compared to WT suggests decreased level of co-regulation among lipid species in the ob/ob mouse liver tissue, which can be attributed to ob/ob organ-specific preferential enrichment of subset of lipids. Confirming PLS/DA results, association of ceramides and triacylglycerols is also observed using correlation network analysis.

**Figure 8:**
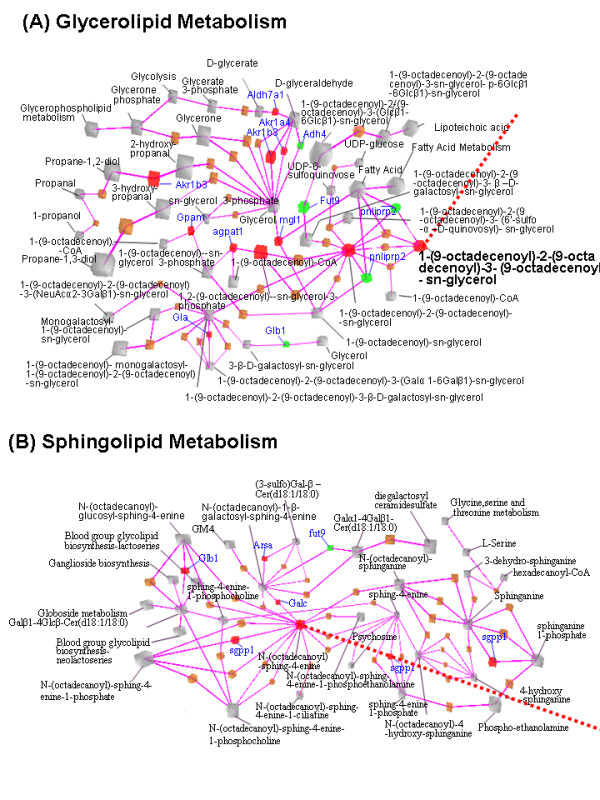
**Instantiated pathways for Cer(d18:1/18:0) and TG(18:1/18:1/18:1)**. Each node represents either lipid metabolite or enzyme or other interconnecting metabolism. Grey color represents metabolites/other metabolism and brown represents enzymes. Up and down regulation of corresponding nodes are denoted by red and green colors, respectively. Enzyme names are shown only if they are differentially regulated (~1.5 fold change). A) Instantiation of TG(18:1/18:1/18:1) as part of glycerolipid metabolism. (B) Instantiation of Cer(d18:1/18:0) as part of sphingolipid metabolism.

We then selected two lipid species, TG(54:3) and Cer(d18:1/18:0) from the Figure 7B, and mapped them into the glycerolipid [51] and sphingolipid [52] reference pathways, respectively (Figure 8). While the notation TG(54:3) is redundant as there may be several corresponding lipid molecular species with the same functional group, total number of acyl carbons and double bonds, we selected one particular instance, TG(18:1/18:1/18:1), for pathway representation. The Figure 8A shows how this particular lipid species is located in the enzymatic system of the glycerolipid pathway. The other pathway, sphingolipid pathway, is instantiated from the co-regulated network with the ceramide lipid species Cer(d18:1/18:0). We utilize for illustration the only publicly available liver ob/ob mouse gene expression data from ChipperDB [53], obtained from 2 month old male mice.

From the sphingolipid pathway map (Figure 8B) two enzymes linked to the ceramide *via *metabolic reactions, one is SGPP1 (Sphingosine-1-phosphate phosphatase 1, UniprotID Q9JI99), the other GALC (galactosylceramidase, UniprotID P54818) were upregulated in ob/ob. SGPP1 is involved in *de novo *ceramide synthesis, while GALC is involved in release of ceramide from glycosphingolipids. Interestingly, sphingomyelin SM(d18:1/18:0) as the known precursor of ceramide via the sphingomyelinase enzymatic action is downregulated, while the sphingomyelinase level is maintained. Therefore, these results indicate that both glycolipids and free fatty acids may contribute as a source of the elevated ceramides observed in the ob/ob fatty liver. The elevated fatty acid flux into the peripheral tissues is a known factor leading to increased ceramide synthesis [50]. In contrast, mobilization of glycosphingolipids for the synthesis of ceramide has not yet been characterized in context of obesity or insulin resistance, although the importance of glycosphingolipids in regulation of insulin sensitivity has been recognized [54]. This is now clearly one area to be investigated further.

## Conclusion

Our lipid informatics strategy greatly facilitated interpretation of ob/ob mouse liver lipidomic profiles which resulted in identification of several lipid molecular species. Notable changes in mean lipid levels comparing obese and their normal littermates among the identified lipids included upregulation of tri- and di-acylglycerol species, diacylphosphoglycerols and specific ceramide species, and downregulation of sphingomyelins in ob/ob mice. Correlation network analysis revealed decreased level of co-regulation among lipid species in the ob/ob condition reflecting the specific enrichment of subset of lipids. We observed associations of short and medium chain triacylglycerols and ceramides, both in ob/ob and WT mice, although these species were significantly upregulated in ob/ob mice. The pathway instantiation of specific lipid molecular species in combination to available gene expression data revealed that both glycolipids and free fatty acids are the sources of elevated ceramides in ob/ob fatty liver.

## Methods

### Database implementation

The lipid data is stored in a native XML database implemented in Tamino XML Server (Software AG). Each compound entry in the database is described by an internal identifier, scoring information, class, canonical SMILES, molecular formula, molecular weight and isotopic distribution. Perl scripts were developed to convert the data into XML documents. Resulting XML documents are loaded using mass-loading tool of the Tamino database.

In the course of implementing the above steps we made use of XMLSPY software (Altova, Inc.) and Tamino Schema Editor Software (Software AG) for the construction and validation of logical and physical schemas, respectively.

### Heuristic lipid database scoring

In order to facilitate database searches, an assigned scoring value for each compound in the database is computed from scoring values of seed fatty acid chains from which that compound is formed. Common factors considered while assigning the scoring to seed fatty acid chains are natural abundance of the fatty acid and odd/even number of carbon atoms present in a fatty acid chain. In addition, different type of bonding (e.g. linked via ether bonds) of fatty acids to glycerol back bone carbon gets different score. The lesser the score the more likely the compound is found in the nature. The total score of a lipid is then a product of all fatty acid scores. Random score S of any lipid compound with one or more fatty acid chains whose score variables V_i _(at Sn1 position), V_j _(at Sn2 position) and V_k_(at Sn3 position) is obtained as follows

S={Vi or VjFor compounds with single fatty acid chains (at sn1 or sn2)Vi×VjFor compounds with two fatty acid chains (at sn1 and sn2)Vi×Vj×VkFor compounds with three fatty acid chains (at sn1, sn2 and sn3)
 MathType@MTEF@5@5@+=feaafiart1ev1aaatCvAUfKttLearuWrP9MDH5MBPbIqV92AaeXatLxBI9gBaebbnrfifHhDYfgasaacH8akY=wiFfYdH8Gipec8Eeeu0xXdbba9frFj0=OqFfea0dXdd9vqai=hGuQ8kuc9pgc9s8qqaq=dirpe0xb9q8qiLsFr0=vr0=vr0dc8meaabaqaciaacaGaaeqabaqabeGadaaakeaacqqGtbWucqGH9aqpdaGabeqaauaabaqadiaaaeaacqqGwbGvdaWgaaWcbaGaeeyAaKgabeaakiabbccaGiabb+gaVjabbkhaYjabbccaGiabbAfawnaaBaaaleaacqqGQbGAaeqaaaGcbaGaeeOrayKaee4Ba8MaeeOCaiNaeeiiaaIaee4yamMaee4Ba8MaeeyBa0MaeeiCaaNaee4Ba8MaeeyDauNaeeOBa4MaeeizaqMaee4CamNaeeiiaaIaee4DaCNaeeyAaKMaeeiDaqNaeeiAaGMaeeiiaaIaee4CamNaeeyAaKMaeeOBa4Maee4zaCMaeeiBaWMaeeyzauMaeeiiaaIaeeOzayMaeeyyaeMaeeiDaqNaeeiDaqNaeeyEaKNaeeiiaaIaeeyyaeMaee4yamMaeeyAaKMaeeizaqMaeeiiaaIaee4yamMaeeiAaGMaeeyyaeMaeeyAaKMaeeOBa4Maee4CamNaeeiiaaIaeeikaGIaeeyyaeMaeeiDaqNaeeiiaaIaee4CamNaeeOBa4MaeeymaeJaeeiiaaIaee4Ba8MaeeOCaiNaeeiiaaIaee4CamNaeeOBa4MaeeOmaiJaeeykaKcabaGaeeOvay1aaSbaaSqaaiabbMgaPbqabaGccqGHxdaTcqqGwbGvdaWgaaWcbaGaeeOAaOgabeaaaOqaaiabbAeagjabb+gaVjabbkhaYjabbccaGiabbogaJjabb+gaVjabb2gaTjabbchaWjabb+gaVjabbwha1jabb6gaUjabbsgaKjabbohaZjabbccaGiabbEha3jabbMgaPjabbsha0jabbIgaOjabbccaGiabbsha0jabbEha3jabb+gaVjabbccaGiabbAgaMjabbggaHjabbsha0jabbsha0jabbMha5jabbccaGiabbggaHjabbogaJjabbMgaPjabbsgaKjabbccaGiabbogaJjabbIgaOjabbggaHjabbMgaPjabb6gaUjabbohaZjabbccaGiabbIcaOiabbggaHjabbsha0jabbccaGiabbohaZjabb6gaUjabbgdaXiabbccaGiabbggaHjabb6gaUjabbsgaKjabbccaGiabbohaZjabb6gaUjabbkdaYiabbMcaPaqaaiabbAfawnaaBaaaleaacqqGPbqAaeqaaOGaey41aqRaeeOvay1aaSbaaSqaaiabbQgaQbqabaGccqGHxdaTcqqGwbGvdaWgaaWcbaGaee4AaSgabeaaaOqaaiabbAeagjabb+gaVjabbkhaYjabbccaGiabbogaJjabb+gaVjabb2gaTjabbchaWjabb+gaVjabbwha1jabb6gaUjabbsgaKjabbohaZjabbccaGiabbEha3jabbMgaPjabbsha0jabbIgaOjabbccaGiabbsha0jabbIgaOjabbkhaYjabbwgaLjabbwgaLjabbccaGiabbAgaMjabbggaHjabbsha0jabbsha0jabbMha5jabbccaGiabbggaHjabbogaJjabbMgaPjabbsgaKjabbccaGiabbogaJjabbIgaOjabbggaHjabbMgaPjabb6gaUjabbohaZjabbccaGiabbIcaOiabbggaHjabbsha0jabbccaGiabbohaZjabb6gaUjabbgdaXiabbYcaSiabbccaGiabbohaZjabb6gaUjabbkdaYiabbccaGiabbggaHjabb6gaUjabbsgaKjabbccaGiabbohaZjabb6gaUjabbodaZiabbMcaPaaaaiaawUhaaaaa@2AE7@

### Animal model background information

Animals were housed at a density of four animals per cage in a temperature-controlled room (20–22*f*C) with 12-h light/dark cycles. Food and water were available *ad libitum*. All animal protocols used in this study were approved by the UK Home Office and the University of Cambridge.

The following genotypes were used for analysis: WT (Lep +/Lep +) and ob/ob (Lep ob/Lep ob). The study included 12 ob/ob (6 male, 6 female) and 10 WT (7 male, 3 female) mice of 16-week age. Genotyping for the point mutation in the ob gene was performed by PCR using standard protocols.

For light microscopy analysis, liver tissues were carefully dissected and fixed in 10% formalin. Tissue was embedded in paraffin and sectioned using a standard microtome (Leica RM2125RT, Leica, UK). Sections were stained with hematoxylin and eosin (H&E) using standard protocols.

### Lipidomic analysis

An aliquot of 20 μl of an internal standard mixture, 50 μl of 0.15 M sodium chloride and of chloroform: methanol (2:1) (200 μl) was added to the weighed (20–30 mg) tissue sample. The standard mixture contains the following lipids: GPCho(17:0/17:0) (10 μg/ml), GPEtn(17:0/17:0) (90 μ/ml), GPCho(17:0/0:0) (320 μg/ml), Cer(d18:1/17:0) (90 μg/ml) and TG(17:0/17:0/17:0) (100 μg/ml).

The sample was homogenized and vortexed (2 min for liver or 15 sec for islets)) and after 1 hour for liver or 20 min for islets standing centrifuged at 10000 RPM for 3 min. From the separated lower phase, an aliquot was mixed with 10 μl of a labeled standard mixture (10 μg/ml GPCho(16:0/0:0-D3), GPCho(16:0/16:0-D6) and TG(16:0/16:0/16:0-^13^C3) and 0.5–1.0 μl injection was used for LC/MS analysis.

Total lipid extracts were analysed on a Waters Q-Tof Premier mass spectrometer combined with an Acquity Ultra Performance LC™ (UPLC). The column, which was kept at 50°C, was an Acquity UPLC™ BEH C18 10 × 50 mm with 1.7 μm particles. The binary solvent system (flow rate 0.200 ml/min) included A. water (1% 1 M NH4Ac, 0.1% HCOOH) and B. LC/MS grade (Rathburn) acetonitrile/isopropanol (5:2, 1% 1 M NH4Ac, 0.1% HCOOH). The gradient started from 65% A/35% B, reached 100% B in 6 min and remained there for the next 7 min. The total run time per sample including a 5 min re-equilibration step was 18 min. The temperature of the sample organizer was set at 10°C.

Mass spectrometry was carried out on Q-Tof Premier (Waters, Inc.) run in ESI+ mode. The data was collected over the mass range of m/z 300–1200 with a scan duration of 0.2 sec. The source temperature was set at 120°C and nitrogen was used as desolvation gas (800 L/h) at 250°C. The voltages of the sampling cone and capillary were 39 V and 3.2 kV, respectively. Reserpine (50 μg/L) was used as the lock spray reference compound (5 μl/min; 10 sec scan frequency).

Lipid identification was performed using tandem mass spectrometry in negative and positive ion mode, as recently described [32].

## Authors' contributions

LY developed the lipid informatics methodology, performed data analyses, and drafted the manuscript. MK developed method for processing of UPLC/MS lipidomics data. GMG performed the experiments with ob/ob and WT animals. TSL performed the lipidomics analysis. AVP coordinated the *in vivo *studies and drafted the manuscript. MO initated the study, performed data analyses and drafted the manuscript. All authors read and approved the final manuscript.

## Supplementary Material

Additional File 1 Table of seed fatty acids. The table lists the fatty acids utilized for the lipid scaffold generation. Click here for file

Additional File 2 Lipid database (LipidDB) contents. The table lists different lipid classes contained in the database utilized in the paper and their sizes in the database. Click here for file
