# Supplementary material for: Bioinformatics strategies for lipidomics analysis: characterization of obesity related hepatic steatosis
Source: BMC Syst Biol. 2007 Feb 15;1:12. doi: 10.1186/1752-0509-1-12 (PMC1839890; doi:10.1186/1752-0509-1-12)
Supplement: Additional File 1 — Table of seed fatty acids. The table lists the fatty acids utilized for the lipid scaffold generation. [file 1752-0509-1-12-S1.pdf]

Bioinformatics strategies for lipidomics analysis: characterization of obesity related hepatic steatosis

Laxman Yetukuri[1], Mikko Katajamaa[2], Gema Medina-Gomez[3], Tuulikki Seppänen-Laakso[1], Antonio Vidal Puig[3] and Matej Orešič[1,*]

[1]VTT Technical Research Centre of Finland, Tietotie 2, FIN-02044, Espoo, Finland, [2]Turku Centre for Biotechnology, Tykistökatu 6, FIN-20521, Turku, Finland and [3]University of Cambridge Department of Clinical Biochemistry, Addenbrooke's Hospital, Hills Road, CB2 2QR, Cambridge, UK

Additional file 1

Title of data: Lipid database contents
Description of data: The table lists different lipid classes contained in the database utilized in the paper and their sizes in the database.

Class	Description	size
Fatty Acyls	Fattyalcohols/aldehydes/carboxylic acids, CoAs	352
Glycerolipids	Mono acyl/alkyl glycerols<br>Diacyl/alkyl glycerols<br>Triacylglycerols	578592
Glycerophospholipids	Both mono and diacyl/alkylglycerophospholipids.Mainly glycerophosphocholines, glycerophosphoethanolamines, glycerophosphoserines, glycerophosphates, glyceropyrophosphates and glycerophosphoglycerols.	190568
Sphingolipids	Sphingoid bases, various ceramides including ceramide phosphoinositols, ceramide phosphocholines, ceramide phosphoethanolamines,N-acylsphingosines, N-acylsphinganines, ceramide 1-phosphates and sulfatides.	7500
Sterols	Cholesterol esters	82
Plasmalogens (glycerophospholipids)	Special class of phospholipids with O-alkenyl ether bonds	181548
