# Supplementary material for: Bioinformatics strategies for lipidomics analysis: characterization of obesity related hepatic steatosis
Source: BMC Syst Biol. 2007 Feb 15;1:12. doi: 10.1186/1752-0509-1-12 (PMC1839890; doi:10.1186/1752-0509-1-12)
Supplement: Additional File 2 — Lipid database (LipidDB) contents. The table lists different lipid classes contained in the database utilized in the paper and their sizes in the database. [file 1752-0509-1-12-S2.pdf]

# Bioinformatics strategies for lipidomics analysis: characterization of obesity related hepatic steatosis

Laxman Yetukuri[1], Mikko Katajamaa[2], Gema Medina-Gomez[3], Tuulikki Seppänen-Laakso[1], Antonio Vidal Puig[3] and Matej Orešič[1,*]

[1]VTT Technical Research Centre of Finland, Tietotie 2, FIN-02044, Espoo, Finland, [2]Turku Centre for Biotechnology, Tykistökatu 6, FIN-20521, Turku, Finland and [3]University of Cambridge Department of Clinical Biochemistry, Addenbrooke's Hospital, Hills Road, CB2 2QR, Cambridge, UK

## Additional file 2

Title of data: Table of seed fatty acids
Description of data: The table lists the fatty acids utilized for the lipid scaffold generation

| Seed number | Systematic Name (LIPID MAPS nomenclature) | SMILES representation | Score | Total No of Carbons |
|---|---|---|---|---|
| 1 | decanoyl | OC(=O)CCCCCCCCC | 1 | 10 |
| 2 | undecanoyl | OC(=O)CCCCCCCCCC | 4 | 11 |
| 3 | dodecanoyl | OC(=O)CCCCCCCCCCC | 1 | 12 |
| 4 | tridecanoyl | OC(=O)CCCCCCCCCCCC | 4 | 13 |
| 5 | tetradecanoyl | OC(=O)CCCCCCCCCCCCC | 1 | 14 |
| 6 | (5-tetradecenoyl) | OC(=O)CCCC=CCCCCCCCC | 2 | 14 |
| 7 | pentadecanoyl | OC(=O)CCCCCCCCCCCCCC | 4 | 15 |
| 8 | (6-pentadecenoyl) | OC(=O)CCCCC=CCCCCCCCC | 4 | 15 |
| 9 | hexadecanoyl | OC(=O)CCCCCCCCCCCCCCC | 1 | 16 |
| 10 | (9-hexadecenoyl) | OC(=O)CCCCCCCC=CCCCCCC | 1 | 16 |
| 11 | (7-hexadecenoyl) | OC(=O)CCCCCC=CCCCCCCCC | 2 | 16 |
| 12 | heptadecanoyl | OC(=O)CCCCCCCCCCCCCCCC | 4 | 17 |
| 13 | (10-heptadecenoyl) | OC(=O)CCCCCCCCC=CCCCCCC | 4 | 17 |
| 14 | (8-heptadecenoyl) | OC(=O)CCCCCCC=CCCCCCCCC | 4 | 17 |
| 15 | octadecanoyl | OC(=O)CCCCCCCCCCCCCCCCC | 1 | 18 |
| 16 | (9-octadecenoyl) | OC(=O)CCCCCCCC=CCCCCCCCC | 1 | 18 |

| | | | | |
|---|---|---|---|---|
| 17 | (11-octadecenoyl) | OC(=O)CCCCCCCCCC=CCCCCCC | 1 | 18 |
| 18 | (9,12-octadecadienoyl) | OC(=O)CCCCCCCC=CCC=CCCCCC | 1 | 18 |
| 19 | (9,12,15-octadecatrienoyl) | OC(=O)CCCCCCCC=CCC=CCC=CCC | 1 | 18 |
| 20 | (6,9,12,15-octadecatetraenoyl) | OC(=O)CCCCC=CCC=CCC=CCC=CCC | 1 | 18 |
| 21 | nondecanoyl | OC(=O)CCCCCCCCCCCCCCCCCC | 4 | 19 |
| 22 | (10-nondecenoyl) | OC(=O)CCCCCCCCC=CCCCCCCCC | 4 | 19 |
| 23 | (12-nondecenoyl) | OC(=O)CCCCCCCCCCC=CCCCCCC | 4 | 19 |
| 24 | (10,13-nondecadienoyl) | OC(=O)CCCCCCCCC=CCC=CCCCCC | 4 | 19 |
| 25 | (10,13,16-nondecatrienoyl) | OC(=O)CCCCCCCCC=CCC=CCC=CCC | 4 | 19 |
| 26 | (7,10,13,16-nondecatetraenoyl) | OC(=O)CCCCCC=CCC=CCC=CCC=CCC | 4 | 19 |
| 27 | eicosanoyl | OC(=O)CCCCCCCCCCCCCCCCCCC | 1 | 20 |
| 28 | (11-eicosenoyl) | OC(=O)CCCCCCCCCC=CCCCCCCCC | 1 | 20 |
| 29 | (13-eicosenoyl) | OC(=O)CCCCCCCCCCCC=CCCCCCC | 2 | 20 |
| 30 | (11,14-eicosedienoyl) | OC(=O)CCCCCCCCCC=CCC=CCCCCC | 2 | 20 |
| 31 | (8,11,14-eicosetrienoyl) | OC(=O)CCCCCCC=CCC=CCC=CCCCCC | 1 | 20 |
| 32 | (5,8,11,14-eicosetetraenoyl) | OC(=O)CCCC=CCC=CCC=CCC=CCCCCC | 1 | 20 |
| 33 | (5,8,11,14,17-eicosepentaenoyl) | OC(=O)CCCC=CCC=CCC=CCC=CCC=CCC | 1 | 20 |
| 34 | heneicosanoyl | OC(=O)CCCCCCCCCCCCCCCCCCCC | 4 | 21 |
| 35 | (12-heneicosenoyl) | OC(=O)CCCCCCCCCCC=CCCCCCCCC | 4 | 21 |
| 36 | (14-heneicosenoyl) | OC(=O)CCCCCCCCCCCCC=CC | 4 | 21 |

| | | | | |
|---|---|---|---|---|
| | | CCCCC | | |
| 37 | (12,15-heneicosedienoyl) | OC(=O)CCCCCCCCCCC=CCC=CCCCCC | 4 | 21 |
| 38 | (9,12,15-heneicosetrienoyl) | OC(=O)CCCCCCCC=CCC=CCC=CCCCCC | 4 | 21 |
| 39 | (6,9,12,15-heneicosetetraenoyl) | OC(=O)CCCCC=CCC=CCC=CCC=CCCCCC | 4 | 21 |
| 40 | (6,9,12,15,18-heneicosepentaenoyl) | OC(=O)CCCCC=CCC=CCC=CCC=CCC=CCC | 4 | 21 |
| 41 | docosanoyl | OC(=O)CCCCCCCCCCCCCCCCCCCC | 1 | 22 |
| 42 | (13-docosenoyl) | OC(=O)CCCCCCCCCCCC=CCCCCCCCC | 1 | 22 |
| 43 | (13,16-docosadienoyl) | OC(=O)CCCCCCCCCCCC=CCC=CCCCCC | 2 | 22 |
| 44 | (13,16,19-docosatrienoyl) | OC(=O)CCCCCCCCCCCC=CCC=CCC=CCC | 2 | 22 |
| 45 | (10,13,16,19-docosatetraenoyl) | OC(=O)CCCCCCCCC=CCC=CCC=CCC=CCC | 2 | 22 |
| 46 | (7,10,13,16,19-docosapentaenoyl) | OC(=O)CCCCCC=CCC=CCC=CCC=CCC=CCC | 1 | 22 |
| 47 | (4,7,10,13,16,19-docosahexaenoyl) | OC(=O)CCC=CCC=CCC=CCC=CCC=CCC=CCC | 1 | 22 |
| 48 | tricosanoyl | OC(=O)CCCCCCCCCCCCCCCCCCCCC | 4 | 23 |
| 49 | (14-tricosenoyl) | OC(=O)CCCCCCCCCCCCC=CCCCCCCCC | 4 | 23 |
| 50 | (14,17-tricosadienoyl) | OC(=O)CCCCCCCCCCCCC=CCC=CCCCCC | 4 | 23 |
| 51 | (14,17,20-tricosatrienoyl) | OC(=O)CCCCCCCCCCCCC=CCC=CCC=CCC | 4 | 23 |
| 52 | (11,14,17,20-tricosatetraenoyl) | OC(=O)CCCCCCCCCC=CCC=CCC=CCC=CCC | 4 | 23 |
| 53 | (8,11,14,17,20-tricosapentaenoyl) | OC(=O)CCCCCCC=CCC=CCC=CCC=CCC=CCC | 4 | 23 |
| 54 | (5,8,11,14,17,20-tricosahexaenoyl) | OC(=O)CCCC=CCC=CCC=CCC=CCC=CCC=CCC | 4 | 23 |
| 55 | tetracosanoyl | OC(=O)CCCCCCCCCCCCCCCCCCCCCC | 1 | 24 |

| | | | | |
|---|---|---|---|---|
| 56 | (15-tetracosenoyl) | OC(=O)CCCCCCCCCCCCCC=CCCCCCCCC | 1 | 24 |
| 57 | (15,18-tetracosadienoyl) | OC(=O)CCCCCCCCCCCCCC=CCC=CCCCCC | 2 | 24 |
| 58 | (15,18,21-tetracosatrienoyl) | OC(=O)CCCCCCCCCCCCCC=CCC=CCC=CCC | 2 | 24 |
| 59 | (12,15,18,21-tetracosatetraenoyl) | OC(=O)CCCCCCCCCCC=CCC=CCC=CCC=CCC | 2 | 24 |
| 60 | (9,12,15,18,21-tetracosapentaenoyl) | OC(=O)CCCCCCCC=CCC=CCC=CCC=CCC=CCC | 2 | 24 |
| 61 | (6,9,12,15,18,21-tetracosahexaenoyl) | OC(=O)CCCCC=CCC=CCC=CCC=CCC=CCC=CCC | 2 | 24 |
| 62 | pentacosanoyl | OC(=O)CCCCCCCCCCCCCCCCCCCCCCCC | 4 | 25 |
| 63 | (16-pentacosenoyl) | OC(=O)CCCCCCCCCCCCCCC=CCCCCCCCC | 4 | 25 |
| 64 | (16,19-pentacosadienoyl) | OC(=O)CCCCCCCCCCCCCCC=CCC=CCCCCC | 4 | 25 |
| 65 | (16,19,22-pentacosatrienoyl) | OC(=O)CCCCCCCCCCCCCCC=CCC=CCC=CCC | 4 | 25 |
| 66 | (13,16,19,22-pentacosatetraenoyl) | OC(=O)CCCCCCCCCCCC=CCC=CCC=CCC=CCC | 4 | 25 |
| 67 | (10,13,16,19,22-pentacosapentaenoyl) | OC(=O)CCCCCCCCC=CCC=CCC=CCC=CCC=CCC | 4 | 25 |
| 68 | (7,10,13,16,19,22-pentacosahexaenoyl) | OC(=O)CCCCCC=CCC=CCC=CCC=CCC=CCC=CCC | 4 | 25 |
| 69 | hexacosanoyl | OC(=O)CCCCCCCCCCCCCCCCCCCCCCCCC | 2 | 26 |
| 70 | (17-hexacosenoyl) | OC(=O)CCCCCCCCCCCCCCCC=CCCCCCCCC | 2 | 26 |
| 71 | (17,20-hexacosadienoyl) | OC(=O)CCCCCCCCCCCCCCCC=CCC=CCCCCC | 2 | 26 |
| 72 | (17,20,23-hexacosatrienoyl) | OC(=O)CCCCCCCCCCCCCCCC=CCC=CCC=CCC | 2 | 26 |
| 73 | (14,17,20,23-hexacosatetraenoyl) | OC(=O)CCCCCCCCCCCCC=CCC=CCC=CCC=CCC | 2 | 26 |
| 74 | (11,14,17,20,23-hexacosapentaenoyl) | OC(=O)CCCCCCCCCC=CCC=CCC=CCC=CCC=CCC | 2 | 26 |
| 75 | (8,11,14,17,20,23- | OC(=O)CCCCCCC=CCC=CCC= | 2 | 26 |

| | | | | |
|---|---|---|---|---|
| | hexacosahexaenoyl) | CCC=CCC=CCC=CCC | | |
| 76 | heptacosanoyl | OC(=O)CCCCCCCCCCCCCCCCCCCCCCCCCC | 4 | 27 |
| 77 | (18-heptaacosenoyl) | OC(=O)CCCCCCCCCCCCCCCCC=CCCCCCCCC | 4 | 27 |
| 78 | (18,21-heptacosadienoyl) | OC(=O)CCCCCCCCCCCCCCCCC=CCC=CCCCCC | 4 | 27 |
| 79 | (18,21,24-heptacosatrienoyl) | OC(=O)CCCCCCCCCCCCCCCCC=CCC=CCC=CCC | 4 | 27 |
| 80 | (15,18,21,24-heptacosatetraenoyl) | OC(=O)CCCCCCCCCCCCC=CCC=CCC=CCC=CCC | 4 | 27 |
| 81 | (12,15,18,21,24-heptacosapentaenoyl) | OC(=O)CCCCCCCCCC=CCC=CCC=CCC=CCC=CCC | 4 | 27 |
| 82 | (9,12,15,18,21,24-heptacosahexaenoyl) | OC(=O)CCCCCCC=CCC=CCC=CCC=CCC=CCC=CCC | 4 | 27 |
